# Factors associated with stunting among children according to the level of food insecurity in the household: a cross-sectional study in a rural community of Southeastern Kenya

**DOI:** 10.1186/s12889-015-1802-6

**Published:** 2015-04-30

**Authors:** Chisa Shinsugi, Masaki Matsumura, Mohamed Karama, Junichi Tanaka, Mwatasa Changoma, Satoshi Kaneko

**Affiliations:** Graduate School of International Health Development, Nagasaki University, Nagasaki, Japan; Department of Human Ecology, Graduate School of Medicine, The University of Tokyo, Tokyo, Japan; Liaison Center for International Education, Nagasaki University, Nagasaki, Japan; Centre for Public Health Research, Kenya Medical Research Institute (KEMRI), Nairobi, Kenya; Department of Eco-epidemiology, Institute of Tropical Medicine, Nagasaki University, 1-12-4 Sakamoto, Nagasaki, 852-8523 Japan; Department of Nursing, Graduate School of Biomedical Sciences, Nagasaki University, Nagasaki, Japan; Nagasaki University Institute of Tropical Medicine (NUITM) - Kenya Medical Research Institute (KEMRI) Project, Nairobi, Kenya

**Keywords:** Child malnutrition, Stunting, Household food insecurity, Africa

## Abstract

**Background:**

Chronic malnutrition or stunting among children under 5 years old is affected by several household environmental factors, such as food insecurity, disease burden, and poverty. However, not all children experience stunting even in food insecure conditions. To seek a solution at the local level for preventing stunting, a cross-sectional study was conducted in southeastern Kenya, an area with a high level of food insecurity.

**Methods:**

The study was based on a cohort organized to monitor the anthropometric status of children. A structured questionnaire collected information on the following: demographic characteristics, household food security based on the Household Food Insecurity Access Scale (HFIAS), household socioeconomic status (SES), and child health status. The associations between stunting and potential predictors were examined by bivariate and multivariate stepwise logistic regression analyses. Furthermore, analyses stratified by level of food security were conducted to specify factors associated with child stunting in different food insecure groups.

**Results:**

Among 404 children, the prevalence of stunting was 23.3%. The percentage of households with severe food insecurity was 62.5%. In multivariative analysis, there was no statistically significant association with child stunting. However, further analyses conducted separately according to level of food security showed the following significant associations: in the severely food insecure households, feeding tea/porridge with milk (adjusted Odds Ratio [aOR]: 3.22; 95% Confidence Interval [95% CI]: 1.43-7.25); age 2 to 3 years compared with 0 to 5 months old (aOR: 4.04; 95% CI: 1.01-16.14); in households without severe food insecurity, animal rearing (aOR: 3.24; 95% CI: 1.04-10.07); SES with lowest status as reference (aOR range: from 0.13 to 0.22). The number of siblings younger than school age was not significantly associated, but was marginally associated in the latter household group (aOR: 2.81; 95% CI: 0.92-8.58).

**Conclusions:**

Our results suggest that measures against childhood stunting should be optimized according to food security level observed in each community.

## Background

Child malnutrition is still one of the most serious health problems in countries in sub-Saharan Africa and South Asia. It is estimated that nearly 3.1 million children die annually either directly or indirectly as a result of malnutrition [1], and approximately 165 million children are affected by chronic restriction of potential growth [2]. Damage to growth in the early years of life is largely irreversible in terms of human capital development [3-5].

Stunting (low height-for-age) is the chronic restriction of a child’s potential growth. Specifically, it refers to children from the ages of 0 to 59 months who are below 2 standard deviations from the median height-for-age determined by the World Health Organization (WHO) Child Growth Standards [6,7]. Along with wasting (low weight-for-height) and underweight (low weight-for-age), stunting is an indicator of undernutrition. As shown in the conceptual model from UNICEF [8,9], causal factors for stunting in children under 5 years old vary with age and are ecologically linked with each other. Among them, environmental factors in households, i.e., household food security and healthy household environment, play important roles in preventing stunting in the longer term [10-13]. The household environment related to child nutrition consists of the perception by caregivers of food insecurity [14], child health and food selection [15-17], and household socioeconomic status [18]. These intra-household environmental factors contribute to the neglect of children’s needs, especially their nutritional status from birth to preschool. Furthermore, the intra-household environment is affected by environmental, cultural, and historical factors in the communities where the mothers live.

Although the proportion of children with stunted growth has declined from 35% in 2000 to 30% in 2008 to 2009 [19], Kenya is one of 34 countries with the highest burden of child malnutrition in the world [1]. This study was designed to examine the influence of household environmental factors on the nutritional status of children under 5 years old in a community with a high level of food insecurity in rural Kenya, with the aim of seeking solutions at the community level to ameliorate the problem of childhood stunting.

## Methods

A cross-sectional study was conducted in Kwale District in the Coast Province of Kenya in 2012, using a cohort nested to the Health and Demographic Surveillance System (HDSS) program, which follows about 50,000 residents periodically, in collaboration with Nagasaki University and the Kenya Medical Research Institute [20]. In this cohort, we recruited children under 5 years old and their caregivers, including non-biological mothers, from households located within a radius of 2.2 km from the Kizibe Health Center, one of the health centers in the HDSS program area. The radius was set in consideration of accessibility for children and their caregivers to the surveys in the nested-cohort study. We took into consideration the estimated sampling size (438 children) for a 2 sample comparison of proportions calculated in the study design stage assuming that 10% of children would become stunted during the observation period and there would be twice as many children with stunting in the comparison group, which has a factor (exposure) with a power of 80% and a significance level of 5% (2-tailed). This cohort program measured several indices, including anthropometric measurements such as height and weight, and asked questions of mothers related to health status and dietary intake. The measurements were to take place 3 times per year between 2011 and 2014. In this cross-sectional study, a structured questionnaire was additionally administered as part of the follow-up surveys of the cohort to investigate the relationship between intra-household environment and child nutritional status. During the survey period in 2012, 653 households were registered within a 2.2-km radius from the health center of the HDSS program; and among them, 516 children less than 5 years old were identified in 360 households.

After carrying out a pre-test to revise the questionnaire for suitability, we conducted interviews of the caregivers by trained local investigators in the Kiswahili language at the health center. The interview required approximately 20 minutes to complete. The structured questionnaire consisted of the following variables: demographic characteristics; socioeconomic status; household food security; child health status, such as breastfeeding behavior and illness in the past 2 weeks including jigger flea (*Tunga penetrans*) infection; caregiver’s perception of child’s growth; and caregiver’s household chores as a proximal factor of availability for child rearing.

The household food security level was measured using the Household Food Insecurity Access Scale (HFIAS) with scores ranging from 0 to 27 by household level [21]. The HFIAS scores obtained from households were categorized into 4 levels of food insecurity, namely, “food secure,” “mildly food insecure,” “moderately food insecure,” and “severely food insecure,” based on the HFIAS guideline [22]. The household socioeconomic status (SES) was parameterized by the principle component analysis (PCA) method using house properties confirmed by the questionnaire: property owned; source of drinking water; type of toilet facility; and type of flooring, wall material, and roof material. The items of household property were selected according to the Demographic Health Survey (DHS) [19]. The score in the first PCA component was used as an asset index of SES status for each household [23]. According to the PCA-based asset index, households were divided into 4 groups; the first quartile SES group was poorest and the fourth quartile SES group was richest in the study area.

For data validation, Cronbach’s alpha coefficient, which is a measure of the internal consistency of a scale, was used to confirm the reliability of the HFIAS and household SES measure. An alpha value of more than 0.7 indicates that the measure is acceptable. Child age was confirmed using his/her maternal and child health (MCH) handbook or by the response from the caregiver if the MCH handbook was not available.

Anthropometric measurement data were obtained from the child cohort dataset. In the child cohort study, height was measured by a length scale (Seca GmbH & Co.Kg, Germany). Weight was measured using trouser for baby weighing scale (G.S.T. Corporation, India) and portable electronic scale (Guangzhou Weiheng Electronics Co., Ltd, China) for babies; and KRUPS Baby Cum Child Weighing Scale (Doctor Beci Ram & Sons [MFG.], India) for children who could stand. For measuring the weight of caregivers a Tanita THD-650 scale (Tanita, Japan) was used.

Chronic malnutrition (stunting) of children was defined as z-score below 2 standard deviations(SD) from the mean for length or height for age according to the Child Growth Standards published by the WHO in 2006 [6]. For this study, those who had a z-score above −2 SD were defined as children who did not have stunting.

We excluded the following children from the analysis: those whose caregivers were unable to answer the questions due to hearing disability; those who were severely sick; and those whose birth date were not appropriate or unclear. Because 72.3% of children in this study were born at home according to our survey data, some birth dates were not clearly recorded.

The association between potential predictors (child and caregiver characteristics, intra-household environment, food intake, and health history) and stunting status was determined by univariate logistic regression analyses. Because some children belong to the same household and may be correlated, cluster options by household were incorporated in the logistic regression. Multiple logistic regression analysis was also conducted to control confounding factors by backward stepwise selection with 0.2 of significant level of removal from the model as well as cluster option by household. Additionally, to identify associated factors of childhood stunting separately in severe and non-severe food insecurity groups, the analyses were independently conducted for the 2 groups in the same statistical manner. Stata statistical software (version 12.0: Stata Corporation, TX, USA) was used for data cleansing and data analyses.

This study was approved by the Ethics Committee of Nagasaki University and authorized as a sub-study of the cohort study by the Ethical Review Committee of the Kenya Medical Research Institute (KEMRI SSC No.1964). Study permission was also obtained from the National Council for Science and Technology (NCST) in Kenya (Research Permit No. NCST/RCD/12A/012/59). We explained the study objectives and obtained written informed consent from all participants before collecting data. Participants were informed that participation in this study was voluntary and that they could stop participating at any time without experiencing negative consequences.

## Results

**Figure 1:**
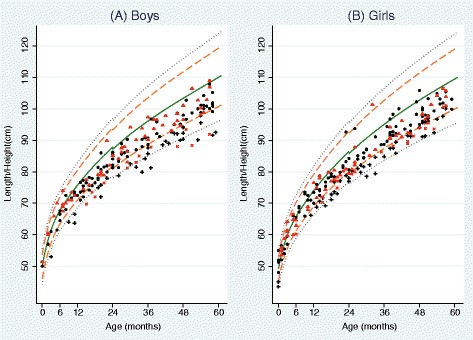
Distributions of length/height-for-age from birth to 59 months: **(A)**: Boys, **(B)**: Girls. The 5 lines represent median (solid green line) and standard deviations (SD) from the median length/height-for-age provided by the World Health Organization Child Growth Standards: +/−3 SD (dotted gray line), +/−2 SD (dashed orange line) between median and +/−3 SD lines. Black circles: children without stunting in the severe food insecure group; black +: children with stunting in the severe food insecure group; red triangle: children without stunting in the non-severe food insecure group; red x: children with stunting in the non-severe food insecure group.

Four hundred twenty-five selected children were initially enrolled in this study. Twenty-one children were excluded (severely sick, 2; no or inadequate date of birth, 17; and hearing disability of caregiver, 2). Finally, 404 children less than 5 years old from 263 households participated in the survey conducted at the health center. The response rate was 78.3% (404/516). Among the remaining children, 94 (23.3%) were stunted and 310 (76.7%) were not stunted according to WHO child growth standards [6]. The distributions of height-for-age of the children are presented in Figure 1 along with the WHO standards.

In Table 1, characteristics of the children and their families are displayed according to stunting status of children and crude odds ratios (ORs). Children aged 12 months and older were significantly more likely to be stunted compared with children less than 6 months old. Forty (21.5%) boys and 54 (24.8%) girls were determined to have stunted growth; but there was no significant difference between boys and girls regarding stunting status. There were 263 caregivers for 404 children and the mean age of caregivers was 29.2 years old (standard deviation [SD]: 7.9; range: 14–72). Children who had adolescent caregivers or caregivers more than 40 years old were more likely to be stunted, but this was not statistically significant. The mean body mass index (BMI) of caregivers was 20.7 kg/m2 (range: 13.9-37.5). Ninety-eight (24.5%) caregivers were underweight (BMI < 18.5 kg/m2), but there was no significant association of childhood stunting according to category of caregiver BMI. Almost half of the caregivers had received a primary-school education, but half had not completed primary school. There was no significant difference in child stunting distribution or association according to caregiver education level. The mean number of siblings of preschool age was 1.9 per household. Children with two or more siblings of preschool age were significantly more likely to suffer from chronic malnutrition (OR: 1.69; 95% CI: 1.01-2.83).

**Table 1:** **Distribution of stunting status by characteristics of children (N = 404)**

**Variables**		**Children with stunting**		**Children without stunting**		**Total**
**Child age (mo)**						
	0-5	3	(7.5%)	37	(92.5%)	40
	6-11	6	(15.8%)	32	(84.2%)	38
	12-23	21	(23.1%)	70	(76.9%)	91
	24-35	21	(29.2%)	51	(70.8%)	72
	36-47	24	(32.0%)	51	(68.0%)	75
	48-59	19	(21.6%)	69	(78.4%)	88
**Child gender**						
	Boy	40	(21.5%)	146	(78.5%)	186
	Girl	54	(24.8%)	164	(75.2%)	218
**Caregiver’s age (y)**						
	18 and younger	5	(38.5%)	8	(61.5%)	13
	19-30	51	(22.1%)	180	(77.9%)	231
	31-40	29	(21.5%)	106	(78.5%)	135
	Above 40	8	(34.8%)	15	(65.2%)	23
	Missing	1	(50.0%)	1	(50.0%)	2
**Caregiver’s BMI**						
	Underweight	28	(28.6%)	70	(71.4%)	98
	Normal	56	(21.0%)	211	(79.0%)	267
	Overweight	7	(30.4%)	16	(69.6%)	23
	Obese	3	(25.0%)	9	(75.0%)	12
	Missing	0	(0.0%)	4	(100.0%)	4
**Caregiver’s education status**						
	Not educated	49	(26.2%)	138	(73.8%)	187
	Preschool	0	(0.0%)	9	(100.0%)	9
	Primary	44	(22.8%)	149	(77.2%)	193
	Secondary	1	(6.7%)	14	(93.3%)	15
**Siblings of preschool age**						
	0 or 1	28	(17.8%)	129	(82.2%)	157
	2 or more	66	(26.8%)	180	(73.2%)	246
	Missing	0	(0.0%)	1	(100.0%)	1
HFIAS* category (food insecurity)						
	Secure	15	(20.0%)	60	(80.0%)	75
	Mildly insecure	6	(18.8%)	26	(81.3%)	32
	Moderately insecure	7	(17.9%)	32	(82.1%)	39
	Severely insecure	66	(25.6%)	192	(74.4%)	258
**Socio economic status (SES)**						
	Poorest	31	(28.4%)	78	(71.6%)	109
	Second	19	(20.7%)	73	(79.3%)	92
	Third	29	(28.7%)	72	(71.3%)	101
	Fourth	15	(15.0%)	85	(85.0%)	100
	Missing	0	(0.0%)	2	(100.0%)	2
**Child health conditions**						
**Diarrhea**						
	No	61	(22.2%)	214	(77.8%)	275
	Yes	32	(25.0%)	96	(75.0%)	128
	Missing	1	(100.0%)	0	(0.0%)	1
**Current jiggers infection**						
	No	59	(20.4%)	230	(79.6%)	289
	Yes	34	(29.8%)	80	(70.2%)	114
	Missing	1	(100.0%)	0	(0.0%)	1
**Ring worm**						
	No	69	(21.9%)	246	(78.1%)	315
	Yes	24	(27.3%)	64	(72.7%)	88
	Missing	1	(100.0%)	0	(0.0%)	1
**Dietary intake in previous 24 h**						
**Plain water**						
	No	11	(20.0%)	44	(80.0%)	55
	Yes	83	(23.8%)	266	(76.2%)	349
**Non-milk liquids**						
	No	51	(21.3%)	189	(78.8%)	240
	Yes	42	(25.8%)	121	(74.2%)	163
	Missing	1	(100.0%)	0	(0.0%)	1
**Tea/porridge with milk in previous 24 h**						
	No	58	(20.4%)	227	(79.6%)	285
	Yes	35	(29.7%)	83	(70.3%)	118
	Missing	1	(100.0%)	0	(0.0%)	1

*HFIAS, Household Food Insecurity Access Scale.

Prior to analyzing the relationship between household food insecurity level and child stunting, internal consistency was evaluated by Cronbach’s alpha obtained for the 9 questions of the HFIAS for 263 households. Cronbach’s alpha was 0.96, which indicates that internal consistency was sufficient for further analysis. A score of 0 indicates that the household does not have food insecurity, whereas a score of 27 indicates that the household has severe food insecurity. The mean HFIAS score was 9.85 (SD: 8.5; median: 9.0), with a range of 0 to 27. Among 263 households, 165 (62.7%) were categorized as having severe food insecurity. The number of moderately and mildly insecure and secure households were 29 (11.0%), 22 (8.4%), and 47 (17.9%), respectively. The association with stunting was not significantly different among the food insecure categories.

Regarding SES, the majority of participants lived in compounds consisting of natural materials with several buildings designated for cooking and housing animals. The household wealth quartile index was determined by estimating asset factors through principle component analysis (PCA). The first component of the PCA, with a 16.1% proportion, was used to determine household socioeconomic status (SES) as a proxy indicator. Cronbach’s alpha obtained from the 27 items measuring SES was 0.71, confirming the reliability of this scale. Compared to the poorest category, there was no significant difference in the prevalence of stunting in the second and third SES categories. However, there was 59% less stunting in the fourth SES category (wealthiest) compared to the poorest category.

The distribution and prevalence of illnesses among the children according to the children’s medical records for the previous 2 weeks are listed in Table 1. Diarrhea and jiggers and ringworm infections were included in consideration of their high morbidity in the area. One hundred twenty-eight children (31.7%) had diarrhea in the previous 2 weeks and 114 (28.2%) suffered from jiggers infection, which is an indigenous disease in resource-poor communities and has not been significantly associated with chronic malnutrition among children (OR: 1.66; 95% CI: 0.99-2.77; p = 0.055). The distribution and prevalence of children with stunting by dietary intake in the previous 24 hours are also presented in Table 1. In the previous 24 hours, the majority of children (n: 349; 86.4%) drank plain water and 163 (40.3%) drank non-milk liquids. One hundred eighteen children (29.2%) had tea/porridge with milk in the previous 24 hours and those children were 1.65 times more likely to have stunting (95% CI: 1.03-2.64; p = 0.036) compared with those who did not have tea/porridge with milk.

### Multivariate analysis

The results from the stepwise multiple regression model of stunted children on household and caregiver variables are shown in Table 2. The following factors remained and were incorporated into the regression model: socioeconomic status (SES), child age in months, animal rearing, number of siblings of pre-school age, having something to drink with milk in the previous 24 hours, and current jiggers infection. HFIAS category was forced into the model to evaluate the effect of food insecurity on child stunting. Among the variables remaining in the model, children in households in the highest SES category had 66% less stunting compared with those in the poorest households (OR: 0.34; 95% CI: 0.16-0.72); however, the food insecurity level (HFIAS category) was not significantly different among these groups. Children between 2 and 3 years old were about 3.5 times more likely to be stunted compared with those aged 0 to 5 months (OR: 3.58; 95% CI: 1.33-20.10 for those aged 24–35 months; OR: 3.43; 95% CI: 1.40-18.98 for those aged 36–47 months). Other factors that had tendencies of associations but were not significant were living with 2 or more siblings (preschool-age) compared with those with fewer than 2 siblings: adjusted OR [aOR]: 1.59 (95% CI: 0.93-2.73); given tea or porridge: aOR: 1.69 (95% CI: 0.98-2.93); and a current jiggers infection: aOR: 1.48 (95% CI: 0.83-2.64).

**Table 2:** **Odds ratios (ORs) for child stunting among the whole child group using univariate and multiple logistic regressions**

**Variables**		**Crude OR**	**95% CI**	**Adjusted OR**	**95% CI**
**Child age (mo)**					
	0-5	Ref.		Ref.	
	6-11	2.31	(0.51 - 10.42)	1.85	(0.52 - 10.89)
	12-23	3.70	(1.02 - 13.45)	2.48	(0.92 - 13.95)
	24-35	5.08	(1.46 - 17.69)	3.58	(1.33 - 20.10)
	36-47	5.80	(1.71 - 19.75)	3.43	(1.40 - 18.98)
	48-59	3.40	(0.96 - 12.02)	2.13	(0.72 - 12.10)
**Child gender**					
	Boys	Ref.		Ref.	
	Girls	1.20	(0.75 - 1.92)	1.52	(0.92 - 2.52)
**Caregiver’s age (y)**					
	18 and younger	2.21	(0.62 - 7.84)		
	19-30	Ref.			
	31-40	0.97	(0.56 - 1.67)		
	Above 40	1.88	(0.78 - 4.53)		
	Missing	-			
**Caregiver’s BMI**					
	Underweight	Ref.			
	Normal	0.66	(0.40 - 1.11)		
	Overweight	1.09	(0.41 - 2.94)		
	Obese	0.83	(0.16 - 4.26)		
**Caregiver’s education status**					
	Not educated	Ref.			
	Preschool	-			
	Primary	0.83	(0.51 - 1.35)		
	Secondary	0.20	(0.02 - 1.64)		
**Siblings of preschool age**					
	0 or 1	Ref.		Ref.	
	2 or more	1.69	(1.01 - 2.83)	1.59	(0.93 - 2.73)
HFIAS* category (food insecurity)					
	Secure	Ref.		Ref.	
	Mildly insecure	0.92	(0.29 - 2.95)	1.02	(0.30 - 3.47)
	Moderately insecure	0.88	(0.32 - 2.38)	0.94	(0.32 - 2.77)
	Severely insecure	1.38	(0.70 - 2.69)	1.17	(0.56 - 2.43)
**Socio economic status (SES)**					
	Poorest	Ref.		Ref.	
	Second	0.65	(0.34 - 1.26)	0.59	(0.29 - 1.17)
	Third	0.80	(0.42 - 1.50)	0.68	(0.33 - 1.41)
	Fourth	0.41	(0.20 - 0.82)	0.34	(0.16 - 0.72)
**Child health conditions**					
**Diarrhea**					
	No	Ref.			
	Yes	1.17	(0.71 - 1.93)		
**Current jiggers infection**					
	No	Ref.		Ref.	
	Yes	1.66	(0.99 - 2.77)	1.48	(0.83 - 2.64)
**Ring worm**					
	No	Ref.			
	Yes	1.34	(0.80 - 2.25)		
**Dietary intake in previous 24 h**					
**Plain water**					
	No	Ref.			
	Yes	1.25	(0.66 - 2.37)		
**Non-milk liquids**					
	No	Ref.			
	Yes	1.29	(0.79 - 2.08)		
**Tea/porridge with milk in previous 24 h**					
	No	Ref.		Ref.	
	Yes	1.65	(1.03 - 2.64)	1.69	(0.98 - 2.93)
**Animal rearing**					
	No	Ref.		Ref.	
	Yes	1.82	(1.06 - 3.11)	1.62	(0.87 - 3.01)

Note: 393 among 404 children with non-missing variables were used for the multivariate logistic regression analysis. For multivariate logistic regression, all variables listed in Table 1 were used and selected by backward stepwise selectin with 0.2 of significant level of removal from the model. All the selected variables were used for calculating adjusted odds ratios (adjusted ORs).

*HFIAS, Household Food Insecurity Access Scale

The factors associated with childhood stunting were also analyzed separately for food insecure household groups and groups that were not food insecure using a stepwise logistic regression model, because different factors would affect child stunting status at different food security levels. The results are shown in Table 3. In food insecure households, the significant factors associated with child stunting were tea or porridge intake (aOR: 3.22; 95% CI: 1.43-7.25), child age (those aged 24–35 months), and socioeconomic status. The factor of jigger infection remained in the model but it was not statistically significant (aOR: 1.84; 95% CI: 0.88-3.84).

**Table 3:** **Adjusted odd ratios for child stunting among separate child groups by household food insecurity level using multiple logistic regression**

**1) Severe food insecure group (N = 252)**			**2) Non-severe food insecure group (N = 108)**		
**Variables**	**Adjusted OR**	**95% CI**	**Variables**	**Adjusted OR**	**95% CI**
**Tea/porridge with milk in previous 24 h**			**Animal rearing**		
No	Ref.		No	Ref.	
Yes	3.22	(1.43 - 7.25)	Yes	3.24	(1.04 - 10.07)
**Non-milk liquids**			**Caregiver’s education status**		
No	Ref.		Not educated/Preschool	Ref.	
Yes	0.50	(0.22 - 1.16)	Primary/Secondary	0.44	(0.16 - 1.26)
**Jigger infection**			**Socioeconomic status (SES)**		
No	Ref.		Poorest	Ref.	
Yes	1.84	(0.88 - 3.84)	Second	0.14	(0.03 - 0.73)
			Third	0.22	(0.04 - 1.08)
**Child age (mo)**			Fourth	0.13	(0.03 - 0.55)
0-5	Ref.				
6-11	2.38	(0.48 - 11.92)	**Siblings of preschool age**		
12-23	1.89	(0.47 - 7.62)	0 or 1	Ref.	
24-35	4.04	(1.01 - 16.14)	2 or more	2.81	(0.92 - 8.58)
36-47	3.16	(0.85 - 11.79)			
48-59	1.59	(0.36 - 6.94)			
**Socioeconomic status (SES)**					
Poorest	Ref.				
Second	0.71	(0.33 - 1.53)			
Third	0.80	(0.35 - 1.85)			
Fourth	0.33	(0.13 - 0.85)			

Note:

OR, Odds Ratio; 95% CI, 95% Confidence Interval; HFIAS, Household Food Insecurity Access Scale.

For multivariate logistic regression, all variables listed in Table 1 were used and selected by backward stepwise selection with 0.2 of significant level of removal from the model. All the selected variables were used for calculating adjusted odds ratios (adjusted ORs).

For non-severe-food insecure group, 33 children aged less than 12 months were removed from the analysis because only 1 stunting condition was found in these categories.

In non-food insecure household groups, children in households with animal rearing had a significant association with stunting (aOR: 3.24; 95% CI: 1.04-10.07). Also, socioeconomic status was significantly associated. The association with stunting for participants in the second, third, and fourth SES (wealthiest) categories was significant compared with the first category (poorest). The factor of siblings of pre-school age remained in the model, but was not significant (aOR: 2.81; 95% CI: 0.92-8.58).

## Discussion

This study was initially designed to evaluate the influence of the intra-household environment, especially food insecurity, on chronic malnutrition or stunting among children under 5 years old in a highly food insecure area of Kenya. This idea was initiated by the conceptual framework of the determinants of child undernutrition presented by UNICEF, which describes the relations of these environmental factors in households to stunting in children [7]. The relationships between household food insecurity and childhood stunting has been reported in some Asian and African countries, as indicated in the framework described [24-26]. However, food insecurity level was not significantly related to child stunting in this study. The discrepant result might be due to the skewed distribution of the households in our study toward the food insecurity side with a narrow range, but the real reason cannot be determined with this study design. Nonetheless, it is important to know associated factors for childhood stunting for both food insecure and food secure household groups from the public health perspective.

In households with severe food insecurity, children who had been given tea/porridge with milk within 24 hours before the survey (in Kiswahili: *Vinywaji vywenye maziwa*) were statistically more likely to have stunting. Even though the question about feeding pattern was only for the 24 hours before the survey, such behavior could reflect daily routines in the household. According to observation of households in the study community, some caregivers were giving tea or porridge with milk to their children instead of a meal. As a result, some children did not have 3 meals a day, making them more vulnerable to stunting compared with those children who were not given tea or porridge as a meal. Some caregivers in households with severe food insecurity did not give such food to their children and those children were more likely to attain a nearly normal growth level. These caregivers can be a good model to optimize feeding practices with the locally available foodstuffs even in food insecure conditions. Further investigation in this matter is important to seek a community-level solution to prevent childhood stunting.

Children in the second and third year of life in the severely food insecure group were significantly more likely to have stunting compared with children 0 to 5 months old (Table 3). The following scenario can be assumed: up to 2 years old, a caregiver or mother gives breast milk mainly or as a complementary food to children. Actually, the proportion of breastfed children among our participants becomes zero by 30 months old from our survey data. As the children grow, the caregiver or mother stops giving breast milk and complementary food and shifts to improper feeding practices like providing tea or porridge with milk as a meal. Children then become chronically undernourished at this age. In this scenario, education focused on caregivers’ feeding habits of complementary food for 2 to 3-year-old children can help prevent childhood stunting [27]. Further studies might be necessary to determine the right types of interventions for each community with the problems of childhood stunting and food insecurity.

The presence of jigger flea infection (tungiasis) might have an effect on child stunting in food insecure households, although in our study it was not significant. Tungiasis is a neglected ectoparasitic disease in resource-poor communities; however, the influence of this indigenous insect on younger children has not been thoroughly examined [28]. To date, only a few studies about the ecological description of jiggers have been conducted in Ethiopia [29], Cameroon [30], Brazil [31], and Nigeria [32]. Indeed, the majority of the children in this study, especially young children, did not wear shoes, which increases their risk of contracting jiggers. Other parasitic infections, like hookworm, which causes malnutrition and anemia, can simultaneously infect children in unhygienic conditions like this study area and contribute to stunting [33,34]. Further investigation on stunting and tungiasis, as well as load reduction of tungiasis, is necessary to help combat stunting [35].

In households in the non-severe food insecure group, animal rearing was significantly associated with childhood stunting. The presence of siblings of pre-school age was not significant, but it was marginally associated. These results may indicate that childhood stunting is affected by caregivers who are less readily available for feeding children on a daily basis. The impact of caregivers’ care for infants and young children has been widely acknowledged [36,37]. Some studies have also reported that limited household resources due to the presence of many children negatively influences their nutritional status [38-40]. Furthermore, since their illiterate elder siblings tend to remain in the house longer, caregivers who have many illiterate children may need to allocate psychological and material resources in the household for them. Therefore, last- or next-to-last-born children are less likely to have sufficient meals. In addition, caregivers may not be able to pay sufficient attention to children under 5 years old due to the need to attend to their own responsibilities. Thus, further long-term research about the conditions of intra-household food access, especially for families with illiterate children, is needed. A consensus regarding the importance of public education for children and family planning for parents is also required because caregivers play an important role in the development of healthy infants and young children. In addition, social support such as having assistants to help caregivers at the household level, is urgently required for adequate child growth [41].

In both groups, children of households in the highest SES category were less likely to have stunting. Some studies have identified socioeconomic inequality as a key factor in chronic childhood malnutrition [42,43]. According to the data from the HDSS in this area, the properties of homes used for calculation of SES by PCA are not very diverse in that a large majority of households had wood and mud walls (85.8%) and earth, dung, or sand floors (88.5%), with at least one plot of family land or family members who owned land (96.8%) [20]. Even though SES was divided into 4 categories by PCA, the range was narrow. This variable, therefore, can be interpreted as a controlling factor of SES to evaluate the relationship between childhood stunting and other factors.

There are several limitations in this study. Seasonal changes in the prevalence of stunted children in rural areas of developing countries have been reported [44,45]. Since this study is based on a cross-sectional design, any potential longitudinal relationships between stunted children and seasonal nutritional environmental changes were difficult to assess. Furthermore, the number of children in the study was not large enough to assess factors associated with stunting when we stratified by factors, e.g., food security level and age group. A larger number of children should be recruited to analyze for factor-stratified associations. Additionally, some feeding practices like giving tea/porridge with milk could not be evaluated adequately in this study. Not only increasing the number of subjects in the study, but also deepening the study contents, e.g., anthropological components, should be necessary.

## Conclusions

A quarter of the children under 5 years old in the study area were found to suffer from chronic malnutrition. In the non-severe food insecurity group, animal rearing and SES were factors significantly associated with chronic malnutrition according to food insecurity level. The number of siblings of preschool age was not significantly associated, but was marginally associated. In the severely food insecure group, tea/porridge with milk and child age were significantly associated with child stunting. In other rural community settings of sub-Saharan Africa, the same situation could be happening. Our results suggest that countermeasures against childhood stunting should be optimized according to evidence observed in each community.
